# Experiences of Alert Fatigue and Its Contributing Factors in Hospitals: Qualitative Study

**DOI:** 10.2196/78676

**Published:** 2026-02-19

**Authors:** Nicki Newton, Adeola Bamgboje-Ayodele, Rowena Forsyth, Amina Tariq, Jing Huang, Ramyasri Yannam, Daniel J Lalor, Andrew James Sobey, Melissa T Baysari

**Affiliations:** 1 Digital Health Human Factors Group, Susan Wakil School of Nursing and Midwifery, Faculty of Medicine and Health, The University of Sydney, Susan Wakil Health Building, Camperdown, Australia, 61 410440476; 2 Discipline of Design, School of Architecture, Design and Planning, The University of Sydney, Sydney, Australia; 3 Cyberpsychology Research Group, Faculty of Medicine and Health, The University of Sydney, Sydney, Australia; 4 Australian Centre for Health Services Innovation and Centre for Healthcare Transformation, School of Public Health and Social Work, Faculty of Health, Queensland University of Technology, Brisbane, Australia; 5 Canberra Health Services, Act Health, Canberra, Australia

**Keywords:** alert fatigue, clinical decision support systems, alerts, hospitals, junior doctors, human factors, digital health

## Abstract

**Background:**

Alerts, a key feature of electronic health record systems, intend to improve patient safety by providing timely information at the point of care. However, many electronic health record systems generate excessive alerts that are not immediately clinically relevant and that contribute to alert fatigue. Despite growing recognition of alert fatigue as a safety concern, clinicians’ experiences of alert fatigue and the broader system-level factors that contribute to it being experienced are not well understood.

**Objective:**

This study aims to use a human factors approach to (1) comprehensively explore how alert fatigue is experienced by junior doctors; (2) identify factors that contribute to experiences of alert fatigue; (3) identify perceived impacts of alert fatigue on employees, organizations, and patients; and (4) identify strategies to reduce alert fatigue in practice.

**Methods:**

Semistructured interviews were conducted with junior doctors working in hospitals across Australia. Data were thematically analyzed using a hybrid inductive and deductive approach, informed by the Systems Engineering Initiative for Patient Safety and an information processing model.

**Results:**

A total of 20 junior doctors were interviewed. Alert fatigue was described to occur at different stages of information processing, including when alerts were not detected, were superficially processed using mental shortcuts, or required excessive cognitive effort to interpret. When alerts were not detected or thoroughly processed, participants more often perceived impacts on patient safety and care quality due to the potential to miss important information. Further, when alerts required excessive cognitive effort, participants frequently reported interruptions, frustration, and time and effort loss as impacts. Factors influencing experiences of alert fatigue were identified in all Systems Engineering Initiative for Patient Safety work system domains, including those related to people, tasks, the environment, tools and technologies, and the organization. Key contributors included the design and clinical relevance of alerts, institutional norms and expectations, and information overload from system alerts as well as other alerts and tasks. Alert fatigue was also described to be experienced differently depending on provider characteristics, such as experiences with and knowledge of alerts, mood, and personality, and organizational factors, including culture, shift type, and time of day.

**Conclusions:**

Alert fatigue is not a binary concept but is instead experienced on a continuum and influenced by interacting individual, technical, and contextual factors. Future research should incorporate clinician self-reports to evaluate experiences of alert fatigue in addition to objective measures. Addressing alert fatigue requires tailored interventions that target its different causes and outcomes. These could include technical and design improvements, changes to organizational practices, and individual customization to reduce experiences of fatigue and accommodate differences in clinicians’ needs.

## Introduction

Clinical decision support (CDS) systems have proliferated in health care, forming a core component of many electronic health record (EHR) systems now used in routine practice [1-3]. Within these systems, alerts represent the primary mode of CDS delivery. Common alerts include those that notify clinicians of potential drug-drug interactions, allergies, and dosing errors and those promoting adherence to best practice care [4,5]. Alerts can interrupt clinicians’ workflows during their interactions with the EHR (interruptive alerts) or passively display CDS through icons or flags that require users to actively seek information out (passive alerts) [6]. While alerts are designed to support clinical decision-making and enhance patient safety by providing relevant information at the point of care, many systems generate excessive alerts that are not immediately clinically relevant [4,7-10].

Alert fatigue is a widely reported concern arising from excessive alerting, but as a concept, it remains poorly understood and inconsistently measured [2,11]. Existing research has largely relied on observable behaviors, such as alert overrides and response times, to infer the presence of alert fatigue, assuming that clinicians experiencing alert fatigue will dismiss alerts without considering their content [12,13]. While some studies suggest that alert fatigue arises when clinicians become cognitively overloaded by alerts [4], others report it to be driven by desensitization that develops following repeated exposure to the same alert over time [14,15].

These inconsistencies highlight the limitations of using behavioral proxies to measure what is fundamentally a *subjective* mental state [16,17]. Observable behaviors cannot fully capture clinicians’ cognitive experiences and offer limited insight into the broader factors that contribute to fatigue [18]. While alert fatigue is frequently identified as a barrier to clinicians’ use of alerts in qualitative studies, these studies are typically concerned with overall alert experiences rather than examining alert fatigue itself and the factors that shape it [19-21]. As a result, definitions of alert fatigue and its perceived causes and impacts remain superficial and lack a systemic perspective.

Human factors methods have been widely applied in health care to understand and address system-level factors that impact care delivery, patient safety, and performance outcomes [22]. These approaches are well suited to investigating complex, dynamic issues like alert fatigue, as they account for the cognitive and behavioral processes that occur during clinicians’ interactions with technologies, within the broader context of the tasks, organizational settings, and environments in which these interactions occur [23,24]. Accordingly, this study aimed to (1) comprehensively explore how alert fatigue is experienced by junior doctors; (2) identify factors that contribute to experiences of alert fatigue; (3) identify perceived impacts of alert fatigue on employees, organizations, and patients; and (4) identify strategies to reduce alert fatigue in practice.

## Methods

### Study Design

This study is reported following the SRQR (Standards for Reporting Qualitative Research) guidelines (Checklist 1) [25]. A qualitative design, using semistructured individual interviews, was selected to explore the subjective and nuanced nature of alert fatigue in depth [26,27].

#### Theoretical Frameworks

The Systems Engineering Initiative for Patient Safety (SEIPS) is a human factors model used in health care settings to identify and address safety concerns [24]. The SEIPS describes the “work system,” which comprises 5 domains, including the *person* (both patients and health care providers) at the center of care, who performs *tasks*, using various *tools and technologies* within a *work environment* under specific *organizational conditions*. Interactions between components of the work system influence the delivery of care and other *process*es, which in turn impact *patient*, *employee,* and *organizational outcomes*. The SEIPS model was used to interpret and present data.

**Figure 1.**
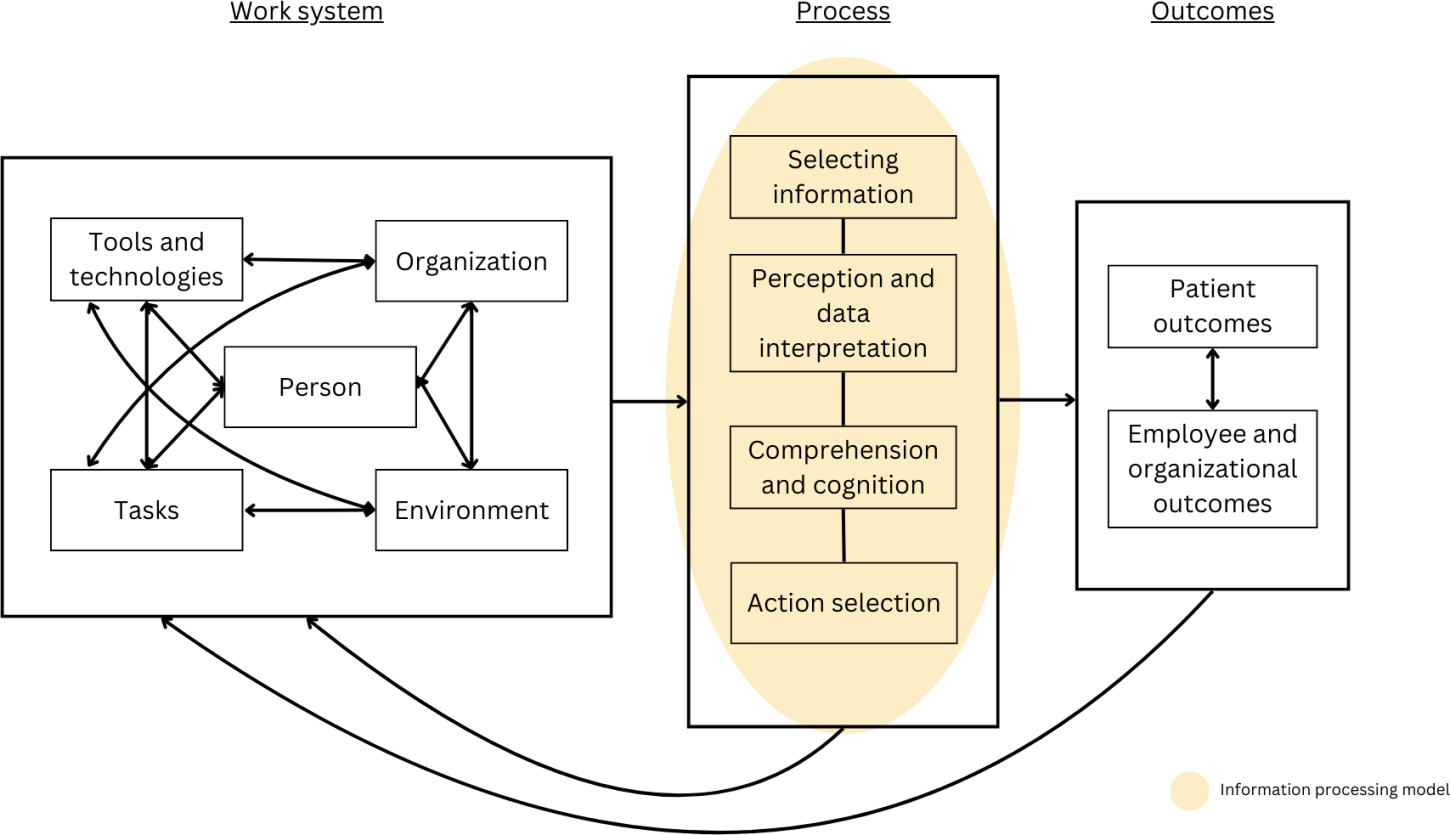
Schematic of the integrated Systems Engineering Initiative for Patient Safety (SEIPS) and information processing models used to interpret data.

We used an information processing model as outlined by Wickens and Carswell [28] to further interpret the *process* by which alert fatigue was experienced by participants. The model describes information processing to occur across 4 stages: *selecting information* (detecting stimuli from the environment), *perception and data interpretation* (recognizing and interpreting the stimuli), *comprehension and cognition* (processing and integrating information with prior knowledge to assess relevance and meaning), and *action selection* (choosing and executing a response based on the processed information). Thus, this model was well suited to understanding the mental processes of alert fatigue and associated actions arising from its presence. Although alert fatigue has previously been described in the context of information processing [29], an analysis of how alert fatigue manifests across different stages has not been explored. A schematic of the integrated models is presented in Figure 1.

#### Study Team

The study team included researchers with expertise across human factors, digital health, health services, sociology, and psychology; and hospital-based pharmacy directors. Additionally, 2 members of the study team were junior doctors, who provided lived experience insights.

### Participants and Recruitment

Eligible participants were practicing junior doctors, also referred to as junior medical officers (JMOs) in Australian hospital systems, defined as interns (postgraduate year [PGY] 1), residents (PGY2), and registrars (PGY3+), who had interacted with alerts in clinical information systems (eg, EHR systems) in hospital settings in Australia. Junior doctors were selected for the study as they are frequent recipients of alerts in hospital settings. A convenience sample of participants was recruited by disseminating details about the study through email distribution lists, posters, and presentations at JMO training sessions, at 2 hospitals within the same hospital network. Additional participants were recruited via snowball sampling through the authors’ professional networks, including word of mouth, LinkedIn posts, and emails to hospital contacts who circulated the study to JMO networks. Prospective participants were encouraged to contact the researchers via email if they were interested in participating.

### Data Collection

A semistructured interview guide was developed using insights from existing research on alert and alarm fatigue and discussion among all authors [4,14,30]. Two pilot interviews were conducted by 1 author (NN) with JMO coresearchers via Microsoft Teams, which resulted in minor refinements to the interview guide, such as adding new questions and changing the order of questions to enhance flow. Questions centered around participants’ use of alerts, perceptions and experiences of alert fatigue, factors influencing alert fatigue, the impact of alert fatigue, and recommendations to reduce experiences of alert fatigue in practice (Multimedia Appendix 1). Individual interviews were conducted with participants by one author (NN) using Microsoft Teams, Zoom (Zoom Communications, Inc), or a phone. Three participants were known to the interviewer prior to the interviews through their professional and personal networks.

Interviews were audio recorded, transcribed using Otter AI software (Otter.ai, Inc) [31], and checked for accuracy. Transcription and analysis occurred in parallel to data collection, allowing for further refinement of the interview guide to explore themes in depth as interviews progressed. Interviews continued until data saturation was reached; that is, no new themes emerged in the final interviews, and only new examples or perspectives related to existing themes were identified [32].

### Data Analysis

Transcripts were imported into NVivo (version 14.24.1; Lumivero) [33] and thematically analyzed using a combined inductive and deductive approach [34]. The first 6 transcripts were independently analyzed by 2 authors (NN and MB or AB or RF) who inductively coded quotes. Themes were deductively mapped to 4 categories: defining alert fatigue, factors influencing experiences of alert fatigue, the impact of alert fatigue, and strategies to reduce alert fatigue. Each pair of authors met to discuss alignment and resolve discrepancies.

Following discussions, 3 of the 4 original categories were restructured under the SEIPS model, where codes related to defining alert fatigue were moved to *processes*, factors influencing experiences of alert fatigue were moved to the *work system*, and the impact of alert fatigue was moved to *outcomes* [24]. Themes related to the process of alert fatigue were further interpreted using the information processing model [28].

Remaining transcripts were analyzed by 1 author (NN) using the SEIPS model, adding new codes and themes as they emerged. Upon completion of the analysis of all interviews, 4 authors (NN, MB, AB, and RF) participated in a workshop to discuss the categorization of codes and themes, which resulted in some codes being renamed, merged, and/or recategorized. The coding framework was then presented to all authors and select participants for member checking, who provided input and agreed upon the final thematic structure.

### Ethical Considerations

Ethical approval was received from the Australian Capital Territory Health Low Risk Ethics Committee (protocol: 2024.LRE.00024). All participants provided written informed consent and did not receive compensation for participating in the study. Interview data were deidentified at the point of transcription, with all potentially identifying details removed. Demographic characteristics are presented in aggregate, and no identifiable participant information is included in this study.

## Results

### Summary

A total of 20 interviews were conducted between August 2024 and March 2025. Participants included 9 interns (PGY1), 6 residents (PGY2), and 5 registrars (PGY3+), working at 10 hospital sites, across 3 Australian states (Table 1). Interviews lasted between 18 and 56 minutes, with an average length of 33 (SD 11; range 18-56) minutes. Two interviews were conducted via phone and the remainder via videoconference.

**Table 1.** Participant characteristics

Characteristics	Participants, n (%)
Role (PGY^a^)	
Intern (PGY1)	9 (45)
Resident (PGY2)	6 (30)
Registrar (PGY3+)	5 (25)
Sex	
Male	12 (60)
Female	8 (40)
Length of time at current hospital	
Less than 2 months	3 (15)
2 months to 1 year	6 (30)
1 to 2 years	4 (20)
2+ years	7 (35)
Clinical experience total (including prior clinical work)	
Less than 2 months	2 (10)
2 months to 1 year	3 (15)
1 to 2 years	4 (20)
2-5 years	5 (25)
5+ years	6 (30)
State	
NSW^b^	11 (55)
ACT^c^	7 (35)
SA^d^	2 (10)
Systems used for alerts	
Commercial	18 (90)
Commercial and homegrown	2 (10)

a PGY: postgraduate year.

b NSW: New South Wales.

c ACT: Australian Capital Territory.

d SA: South Australia.

Participants received alerts from EHR, electronic medical record (EMR), and/or electronic medication management systems. All participants were users of commercially available EHR or EMR systems (eg, Epic, Cerner, Sunrise, and MedChart), while 2 participants also used homegrown systems. Alert types included passive and interruptive alerts such as those for medications (eg, allergy, drug-drug interactions, and dosing), and best practice (eg, venous thromboembolism prophylaxis, advanced care directives, and resuscitation plans). Alerts popped up during tasks such as medication ordering, opening patient charts, and opening the EHR system. Alerts outside of standard clinical information systems (“system”) were also described.

**Figure 2.**
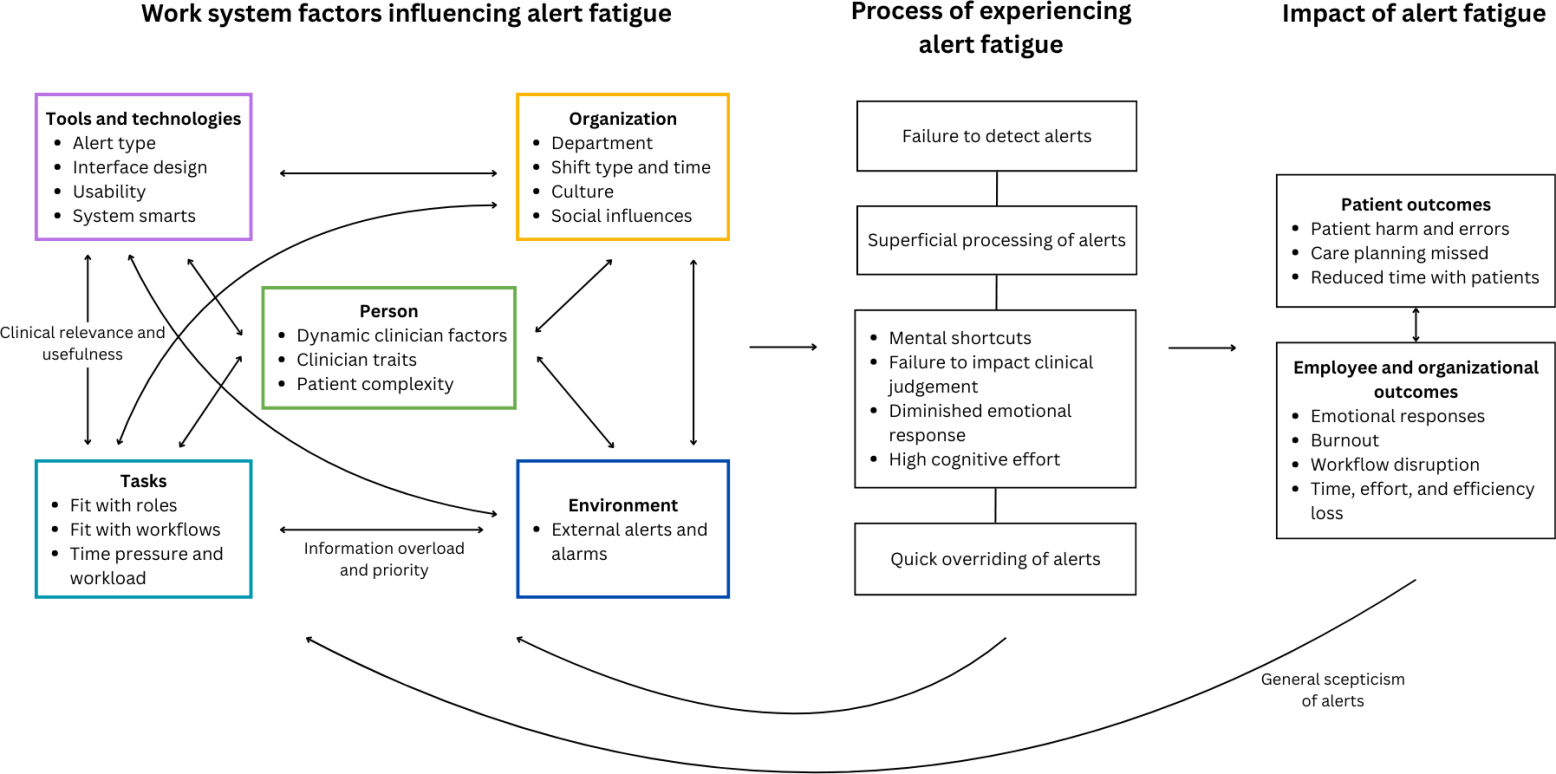
Schematic of themes related to experiences of alert fatigue within the Systems Engineering Initiative for Patient Safety (SEIPS) model.

In the following sections, we present the clinicians’ perceptions of alert fatigue (process), contributing factors (work system), impacts (outcomes), and potential strategies for addressing alert fatigue. Figure 2 shows a schematic of themes aligned to the SEIPS model.

### The Process of Experiencing Alert Fatigue

#### Selecting Information

Alert fatigue manifested during the information selection stage as a failure to notice alerts or detect alert stimuli. This occurred mainly for passive alerts, which were often described to be inconspicuous, but also for interruptive alerts: “The idea of alerts being there is just part of the job, it’s just part of the furniture…I probably at least once will miss the fact that [medications are] expiring, my eyes will gloss over it” (P1).

#### Perception and Data Interpretation

Perception and interpretation involved recognizing and processing alerts. Clinicians described superficially processing alerts by only partially viewing or reading them before responding. Participants explained that alerts were often pre-empted based on their interactions with the system before an alert was triggered or identified by design features such as titles, keywords, fonts, colors, and layouts. Many participants felt that they could quickly and effectively recognize these alerts and often did not feel “fatigued” by alerts that were processed in this manner: “it’s so mindless….All you process is, it’s an ECG one, cancel, it’s an ACD one, cancel” (P14).

#### Comprehension and Cognition

Comprehension and cognition involved assessing the relevance of alerts to inform decision-making. While participants initially described reading and actively interpreting the value of alerts, alert fatigue occurred where active interpretation of alerts and influence on clinical judgment ceased and mental shortcuts instead guided participants:

I would have read the alert the first time, but then…you go, “okay, I know what this alert is about,” and the next day you would have forgotten which drugs are even mentioned. I just know that this is not relevant to me.

[P13]

Emotional responses to alerts, such as anxiety and stress, were reported to be common early on. However, this diminished over time as users reported becoming desensitized and “complacent.” Conversely, some clinicians reported experiencing alert fatigue due to the cognitive effort and time required to interpret and appropriately respond to alerts.

#### Action Selection

Action selection comprised users’ responses to alerts. Alerts that were quickly and habitually overridden signified alert fatigue at this stage. For example, participants described being “so used to clicking boxes*”* (P6) that “you just click through them automatically” (P13) and that “it’s like whack-a-mole. You’re just getting rid of everything as fast as you can” (P14).

Some participants said that they broke habitual responding patterns, for example, when their role changed, or they made an error due to experiencing alert fatigue. However, these patterns were likely to return: “after a while, you desensitize yourself again after that going well, it hasn’t happened for a long time, and your brain kind of becomes complacent again, with going Yes, yes, yes, through all the tick boxes*”* (P18). Some alerts that were quickly overridden were however sometimes still perceived to be useful as they prompted consideration and did not take excessive cognitive effort to process.

### Work System Factors Influencing Experiences of Alert Fatigue

Factors were coded to all work system domains of the SEIPS. Multimedia Appendix 2 shows the distribution of coded references within each domain.

#### Person

Patients’ clinical complexity was reported to influence alert fatigue. Some participants described paying closer attention to alerts for unstable patients, while others described increased fatigue due to the volume of alerts that complex patients triggered.

Clinician traits, including clinician type, clinical experience, and personality, also influenced alert fatigue. While many participants believed that senior clinicians experienced greater fatigue, more senior participants reported experiencing less fatigue than when junior, as they had more confidence and consequently used less cognitive effort to appropriately respond: “when I was more junior and didn’t quite understand as well, I would often just go, ‘advice ignored,’ without really understanding the reason why*”* (P13). Personality traits were also raised, where participants who were more meticulous described paying more attention to alerts: “for me personally, I don’t like just dismissing alerts without actually thinking through it” (P19).

Alert fatigue was influenced by dynamic factors that evolved based on individuals’ experiences with alerts over time. Those who possessed a positive attitude toward alerts generally reported experiencing less fatigue:

I just view these alerts as another opportunity to make sure you have the whole picture.

[P10]

*if you think it’s a pain in the ass, then it’s something that’s going to irritate you every time. But if your attitude is, which mine is, is- “it’s just part of the job,” so you just get on with it.*

[P15]

Alert fatigue was influenced by participants’ knowledge and perceived importance of the risk the alert was targeting, including personal experiences of missing information or making errors: “if I don’t understand or agree with the importance, I’m much more likely to dismiss it with so many other competing priorities” (P17). Additionally, some clinicians explained they did not know how to correctly respond to alerts:

no one told me how to…no one knows. So you just choose something that makes sense*.*

[P2]

Others described remaining cautious and attentive to alerts due to their awareness of alert fatigue.

Alert fatigue was also reported to be influenced by clinicians’ emotional state, including their mood and whether they were experiencing other forms of fatigue: “you feel more emotionally drained, less sympathetic, less compassionate, and you might dismiss things more” (P1).

#### Tasks

Alert fatigue was influenced by workflow alignment, clinical relevance, fit with roles and responsibilities, time pressure, and information overload. Fatigue was often reported to occur where alerts were misaligned with organizational and decision-making processes. Poorly timed alerts, such as those triggered upon opening a patient’s chart, were typically reported to be quickly dismissed: “I intend to view their chart with a very specific goal and updating the resuscitation right then is not a priority” (P16).

Clinical relevance and usefulness relative to clinicians’ roles and responsibilities were often mentioned. While some alert types were seen as entirely irrelevant, others were inconsistently useful. Fatigue also arose where relevant alerts were lacking, forcing users to manually search for information: “it has taken me six clicks to get one answer, which is highly inefficient” (P16). Useful alerts were described to help with cognitive unloading, save time, and provide unfamiliar or easily forgotten information:

*doctors would really appreciate if there was one alert saying all the blood tests that have been ordered are now back…because we spend a significant portion of our cognitive time in the day just thinking about that*.

[P16]

Interestingly, some participants described experiencing less fatigue from alerts with poor utility: “we do not get effective alerts from EMR at the moment, so we don’t rely on those alerts, so there’s no fatigue involved there” (P16).

Alerts perceived to be well aligned with roles and responsibilities often did not induce alert fatigue even when they were often disregarded. This was particularly evident where clinicians had significant individual responsibility: “I’m always very grateful to get [discharge medications] alerts because the consequences of getting something wrong in that way can be disastrous, and it is on you legally” (P1).

In contrast, alerts received by a team of clinicians created a diffusion of responsibility, which led to these alerts being deprioritized: “if it was sent to everyone, it’s everyone’s job” (P17). Participants also described automatically clicking through alerts where they had lower autonomy: “someone’s told you to chart that med, so you just do it*”* (P13).

Situations in which clinicians faced greater time pressure and higher workloads, such as during ward rounds, were described to trigger alert fatigue even in participants who were typically unaffected:

*I don’t feel like I’m at a stage where any alert that pops up- it’s tapped into that alert fatigue. For me, it sort of comes in waves when I’m quite busy within the hospital and there’s a lot of tasks that have built up.*

[P10]

Information overload also contributed, with participants feeling overwhelmed by excess information or many alerts received at once:

*it’ll often give you up to 10 different alerts [in a single encounter]…it’s hard to tell which ones are actually worth stopping for and thinking about, and which ones you just ignore*.

[P4]

Other information presented on screen, such as task lists and message functions within the system, also contributed to overload.

#### Environment

Only one factor was coded to the work environment, which included information overload from *multisensory* nonsystem alerts or individual clinicians’ digital ecosystem, including pagers, emails, WhatsApp messages, auditory alarms, phone calls, and in-person prompts. System alerts were perceived as follows:

*yet another thing that the junior doctor is responsible for or is bombarded with…there’s just so many different ways to message a JMO about a task, or to put a task somewhere for them to do, to page us, to come and find us on the ward…there seems to be a lot of over stimulation.*

[P4]

The level of priority given to system alerts over other tasks was often reported to be low: “with the assumption that if it’s automated, then it’s not as pressing” (P17).

#### Tools and Technologies

Alert type, interface design, system usability, and volume influenced alert fatigue. Participants typically described alert fatigue to develop for specific types of alerts. However, similar-looking alerts could cause confusion or require extra cognitive effort to interpret: “allergies and drug-drug interactions often pop up looking the same, and it takes a little bit longer to work out which one it is” (P10). Participants described interface design features, including the use of colors, font, position, and layout, that could draw their attention; however, a participant stated:

*once you get used to a certain font or format or typeface, the same problem will just happen. Regardless of whether it’s in red or rainbow or purple, once you’ve had it pop up 20 times in one day…you just stop paying attention to it.*

[P13]

Alerts with excess words and jargon contributed to alert fatigue as it could “blur what you’re actually trying to pick up on in the writing as important” (P18), versus shorter alerts which were quickly processed: *“*the alert I pay most attention to is one that’s about 10 words, regardless of the style, if it’s short and something that your brain can process in three seconds, then it is probably going to be read*”* (P13).

Requiring override reasons was perceived positively for important alerts, forcing clinicians to “skim them because…you can’t just say ignore. You’ve got to say, why you ignored, so they force you to put a little bit of accountability against it” (P14). Alert importance was sometimes interpreted based on the presence or absence of additional steps to dismiss the alert: “if it was so important, you couldn’t click x, right?...I think everyone, whether subconsciously or consciously, knows that if it was the be all and end all, there wouldn’t be a not now button” (P20).

Poor usability of alerts and clinical information systems, such as systems being “slow” and “clunky,” was reported to contribute to alert fatigue by disrupting workflows. Clinicians also described interruptions where alerts blocked access to other areas of the system: “I need to cancel that order, stop what I’m doing, go into the allergy section and then interpret that information” (P10).

Alerts lacking system “smarts,” for example, those flagging allergy and drug-drug interactions for patients who were already prescribed the drug in question, were described to contribute to fatigue. Similarly, constant repetition was perceived to create a *“*boy who cried wolf*”* (P17) effect. While an excessive volume of alerts was mentioned, it was only described as problematic where alerts were repetitive, irrelevant, or where multiple alerts were received at once.

#### Organization

Organizational factors included department, shift length and type, institutional culture, social influences, and team dynamics. While experiences of alert fatigue were generally consistent between departments and rotations, differences sometimes arose due to variations in the workload, time pressure, types of tasks and alerts received, and autonomy over tasks. Fatigue was described to increase on after-hours shifts, due to time pressure and the need to cover multiple wards, and at certain times of the day, including late at night. Participants also cited increased alert fatigue at the end of a shift due to workload demands and at the very beginning of a shift where they had insufficient information to appropriately respond.

Institutional and departmental culture and peer behaviors shaped role expectations, which in turn impacted alert fatigue. Alerts aligned with institutional norms were not necessarily less frustrating but were reported to be more widely accepted. Expectations of roles were described to be learned informally from colleagues:

You rock up and people just start asking you to do things…if you haven’t been asked to do it, especially by a senior doctor on your team, then it’s probably not your job.

[P17]

This was reinforced by observing colleagues dismissing alerts:

*they’re like, “Oh, this is so annoying.” Click, click, click. It sets the scene of “Oh, this is just such an inconvenience.” Skip, skip, skip*.

[P8]

New JMOs reported asking colleagues and senior doctors for advice on how to respond to alerts. Alert fatigue was heightened due to the “unspoken pressure about the efficiency that you’re required to have as a junior” (P4), where alerts were perceived to decrease efficiency.

### Impact of Alert Fatigue

#### Employee and Organizational Outcomes

Alert fatigue was associated with feelings of frustration and annoyance, and along with other work stressors, could contribute to burnout and general fatigue. Some participants additionally described guilt; for example, “you just feel as though maybe you’re not doing as good a job…you hate to think that you’re not giving 100% to your patients” (P15). While alert fatigue was often tied to specific alerts, some participants developed broader skepticism, feeling “suspicious of alerts in general” (P6), creating a “sense of mistrust in the system” (P9) after encountering many alerts that were not relevant.

Some participants felt that fatigued users might miss opportunities to streamline their workflows. However, excess alerts were more often described to disrupt workflows, resulting in a loss of time, cognitive effort, and efficiency: “when I’m already holding six pieces of information in my head, then I might lose two, which is the most frustrating aspect” (P9). These interruptions were perceived to impact patient flow (eg, “if things don’t move at a certain speed, the system breaks down, or patients get stuck somewhere, or miss out on discharging home at a particular time*”* [P4]), lead to mistakes or unnecessary responses, and shift attention away from core issues (eg, “it leads to uncertainty, and I think often clouds the issue that the patient’s in hospital for” [P9]).

#### Patient Outcomes

Participants felt alert fatigue could increase the risk of errors and result in actual patient harm, particularly when important alerts were missed or misinterpreted. For example, some participants observed doses being missed, patients being prescribed medications they were allergic to, and pre-emptive care planning being missed. Alert fatigue was also perceived to reduce time spent with patients and inadvertently impact care by reducing clinicians’ patience.

### Strategies to Reduce Alert Fatigue

Strategies to reduce alert fatigue included technical changes such as improving the interface design, personalizing and increasing the relevance of alerts; organizational strategies such as monitoring and evaluation initiatives and educating clinicians about alert fatigue; as well as personal strategies such as improving overall well-being and using workarounds. A full list of strategies is presented in Multimedia Appendix 3.

## Discussion

### Principal Findings

This study provides a comprehensive exploration of junior doctors’ experiences of alert fatigue in hospital settings. Importantly, alert fatigue was found to manifest at different stages of information processing where participants failed to detect alerts, superficially processed alerts, developed mental models, and used excessive cognitive effort to interpret alerts, which led to different response patterns. These experiences were perceived to impact patient safety, such as increasing the risk of errors and contributing to patient harm, and clinician well-being, such as workflow interruptions and reduced efficiency.

The SEIPS model highlighted complex interactions between factors contributing to experiences of alert fatigue, including alert logic and design, organizational culture, and individual traits and emotional states. We observed junior doctors at the same hospital, who received similar alerts, to experience alert fatigue differently depending on their experience, role, and personality. For example, where junior doctors had positive attitudes and alerts were tightly aligned with responsibilities, even frequently disregarded alerts were not perceived as fatiguing. While poor clinical relevance remains a fundamentally important factor in the development of alert fatigue, our study reveals that doctors may have different levels of tolerance depending on these personal, organizational, and task factors. We also observed feedback loops between factors contributing to alert fatigue and its outcomes. For instance, repeated exposure to low-value alerts could lead to negative attitudes, subsequently reducing trust and influencing attitudes toward future alerts.

### Expanding Definitions of Alert Fatigue

In our study, alert fatigue was rarely experienced in an all-or-nothing fashion. Instead, participants often described alert fatigue to arise in specific contexts, such as when under time pressure or for certain alert types. While some participants reported generalized negative perceptions of alerts, none described being universally fatigued or entirely unaffected. Similar to existing studies, we also found alert fatigue to develop for both passive and interruptive alerts [35].

Our findings support the view that alert fatigue is not simply an action, but a mental state that is subjectively experienced at conscious and subconscious levels [16,29]. Participants described 3 distinct information processing pathways in which alert fatigue occurred: one where alert stimuli were not perceived at all, another in which alerts were recognized but superficially processed based on mental shortcuts, and a third where alerts required high cognitive effort to interpret and respond to. The first 2 pathways, in line with existing conceptualizations of alert fatigue, were often associated with alerts being rapidly dismissed [4,12,13]. Despite this, mental shortcuts were perceived as an efficient way to process information and were often not associated with conscious experiences of fatigue. While many participants believed they were able to rapidly interpret alerts, patient safety risks could however still arise due to the potential to misinterpret information, particularly when the alerts lacked visual distinctiveness.

In contrast, the third pathway was consistently described as fatiguing. Participants reported feeling frustrated due to the cognitive burden of alerts and related impacts on efficiency, which arose particularly where alerts were ambiguous, time-consuming, or difficult to action. For certain alert types or individuals, this burden was described to gradually lead to cognitive disengagement, with clinicians eventually defaulting to rapid override as a strategy to avoid further mental effort. This finding mirrors alarm fatigue literature that suggests fatigue develops as a coping mechanism for cognitive burden [30,36]. While burnout is frequently linked to alert fatigue [37], our findings suggest that rapidly overriding alerts may conversely serve as a protective mechanism to manage cognitive load and avoid burnout, consistent with literature linking longer EHR use to increased fatigue and burnout [38,39].

These findings suggest that override rates and response times alone are insufficient measures of alert fatigue. Aligning with prior critiques, our work confirms that frequently overridden alerts are sometimes still attended to and can be perceived as helpful [13,40]. Similarly, although quick dismissals can indicate alert fatigue, they can also represent efficient pattern recognition, while longer interactions could both indicate appropriate engagement with alerts and signal cognitive burden. We therefore recommend supplementing system metrics with clinician self-reports to better understand both conscious and subconscious experiences of fatigue and applying solutions that appropriately address the specific type or types of fatigue experienced.

### Work System Factors Contributing to Alert Fatigue

In line with existing literature, we found exposure to clinically irrelevant, repetitive, and poorly timed alerts contributes to alert fatigue [4,15,21,41,42]. Consistent with quantitative studies, the total volume of system alerts was rarely identified as a cause of alert fatigue alone, but instead became problematic when clinicians received many clinically irrelevant and repetitive alerts, and multiple alerts within a single clinical encounter [4]. We also identified novel influences, including personality traits, mood, social learning, and exposure to external alerts. Many participants described cognitive overload as a result of not just system alerts, but also notifications from pagers, emails, phone calls, and verbal prompts, which cumulatively contributed to experiences of fatigue and dismissal of system alerts. While existing literature has sought to improve total alert burden to reduce alert fatigue [43,44], our study highlights the need to consider system alerts within the broader ecosystem of alerts and tasks that clinicians receive.

Social and organizational factors are underexplored in existing alert fatigue literature but were found to collectively shape experiences in our study. Junior doctors often looked to peers to guide their own responses to alerts and expectations of their roles, affecting not just alert responses but also the framing of alerts as helpful or annoying. Organizational and cultural expectations of efficiency further increased alert fatigue, as managing alerts was often seen as a barrier to achieving other clinical tasks. While alerts are likely to offer the most benefit to junior doctors, our study substantiates prior research that suggests this group experiences more fatigue than their senior counterparts [45]. Participants with more clinical experience had greater confidence and therefore could interpret and respond to alerts with less cognitive effort, whereas those with less experience reported more ambiguity and higher effort expended in interpreting information.

### Recommendations for Addressing Alert Fatigue

Our results highlighted several strategies for reducing alert fatigue that align with prior literature, including removing ineffective alerts and increasing clinical relevance [46], incorporating contextual awareness [47], applying human factors design principles [48,49], and implementing alerts in line with the 5 rights of CDS (right information, person, format, channel, and time) [6,7,50,51]. Our findings build on this literature by providing insight into how and why strategies are likely to be effective, and how they can be targeted to the different causes and impacts of alert fatigue. Several studies testing these strategies have reported their effectiveness in decreasing the overall volume and increasing the acceptance rate of alerts [50]. However, we argue that future research must also evaluate whether clinicians’ *experiences* of fatigue are reduced. Given that our results showed junior doctors are likely to experience greater levels of alert fatigue than more senior doctors, we emphasize the importance of including this group in redesign efforts and evaluation of fatigue reduction.

We also identify and present new strategies that could improve experiences of alert fatigue. These include allowing clinicians to personalize alerts, education on alert fatigue, supporting overall emotional well-being, and streamlining the system and other alerts. Due to the individual variability in experiences of alert fatigue, personalization may be particularly valuable in fostering a greater sense of control and responsibility, thus improving engagement with alerts that individuals deem to be relevant. Further research is needed, however, to evaluate the effectiveness of personalization and other strategies in reducing alert fatigue and improving patient safety.

Interestingly, in some cases, experiences of fatigue were not associated with the presence of alerts, but by their absence, where critical information was difficult and time-consuming to locate. This finding underscores the need for balance between over- and underalerting, ensuring alerts effectively address the clinical or safety need. Implementers should carefully consider the issue they aim to target, evaluating whether CDS and alerts specifically are an appropriate solution, or if different strategies or forms of CDS, such as passive alerts or search functionality, should instead be considered [3,6].

### Limitations

Our participants were junior doctors in urban Australian hospitals who primarily used commercial information systems. Findings therefore may not generalize to other clinician groups, specialties, or settings, such as rural hospitals or those using homegrown systems. Additionally, we did not incorporate observational or system usage data, which may limit insights into subconscious behaviors. Future studies should incorporate mixed methods, that is, both clinicians’ experiences of alert fatigue and actual responses to alerts and explore experiences of alert fatigue among different user groups.

To our knowledge, participants in our study used only knowledge-based alerts, limiting conclusions regarding experiences of alert fatigue for smarter alerts, such as those that are artificial intelligence (AI)-based. Our results can, however, offer important considerations. Although AI-based alerts can significantly improve clinical relevance and specificity [52], they may also increase cognitive burden if information is complex or constantly changing. Alternatively, given the fast-paced nature of the setting in which alerts are presented, AI-based alerts may be superficially processed and, due to their high positive predictive value, be automatically accepted rather than rejected due to automation bias [53]. Further research is needed to explore how alert fatigue manifests for smarter alerts in hospital settings and its impact on patient safety.

### Conclusions

This study highlights alert fatigue as a dynamic, context-dependent experience that is not easily captured through studying behavior alone. Our findings reinforce the need to move beyond using singular metrics to measure alert fatigue and instead address alert fatigue using tailored strategies that target the specific causes and outcomes involved. While reducing low-value alerts and improving interface design remain important, broader approaches that support clinician well-being, foster positive organizational culture, and consider the impact of competing priorities and tasks may be equally critical.

## Supplementary material

10.2196/78676 Multimedia Appendix 1 Semistructured interview guide.

10.2196/78676 Multimedia Appendix 2 Proportion of coded references related to alert fatigue mapped to each Systems Engineering Initiative for Patient Safety (SEIPS) work system domain.

10.2196/78676 Multimedia Appendix 3 Strategies discussed by study participants for reducing alert fatigue in practice.

10.2196/78676 Checklist 1 SRQR checklist.

## Abbreviations

AI: artificial intelligence
CDS: clinical decision support
CRC: Cooperative Research Centre
EHR: electronic health record
EMR: electronic medical record
JMO: junior medical officer
PGY: postgraduate year
SEIPS: Systems Engineering Initiative for Patient Safety
SRQR: Standards for Reporting Qualitative Research
