# Supplementary material for: Experiences of Alert Fatigue and Its Contributing Factors in Hospitals: Qualitative Study
Source: J Med Internet Res. 2026 Feb 19;28:e78676. doi: 10.2196/78676 (PMC12919987; doi:10.2196/78676)
Supplement: Multimedia Appendix 1 [file jmir-v28-e78676-s001.docx]

## Interview Guide

**Use of alerts**

1. What system/s do you typically receive alerts from?
2. What type of alerts do you receive?
   1. Can you give me an example of what you do that triggers an alert?
   2. How do you typically respond to alerts? What influences your response/s?
   3. On average, how many alerts would you usually receive per shift?
3. In general, what do you think of the alerts you receive? (prompt: if positive, why/why not)
4. Thinking back to your first encounter/s with an alert…
   1. What were your first impressions of the alerts you received?
   2. How did this change over time?

**Alert fatigue**

1. How would you define alert fatigue?
2. How would you measure alert fatigue?
3. Do you experience alert fatigue? Explain why/why not

- If no… Given you don’t experience alert fatigue, what helps you to avoid it?
- If yes…

1. How long ago did you start experiencing alert fatigue?
2. Do you remember how your alert fatigue developed?
3. How long did it take to develop?
4. What does alert fatigue feel like? How do you know you’re experiencing it?
   - 1. Any psychological response?
     2. Any physical response?
     3. What is the consequence of this feeling?
5. What contributes to your experience of alert fatigue? Prompts:
   - 1. Work complexity (/patient complexity)
     2. Workload
     3. Relevance/accuracy of alerts
     4. Exposure to alerts
     5. Desensitisation over time
     6. Personal factors e.g. mood etc.
6. Is your experience of alert fatigue different for:
   - 1. Different systems?
     2. Different departments/hospitals?
     3. Different types of alerts?
     4. Different patients?
     5. Different contexts? E.g. ward rounds, after hours
7. How does your alert fatigue impact you and your work?
   1. How does alert fatigue affect patient care or outcomes?
   2. Have you ever missed or dismissed an alert that turned out to be important? Can you describe what happened?
8. What strategies do you take to reduce your alert fatigue?
   1. Do you have any recommendations for how alerts could be improved to reduce alert fatigue? Prompts:
      1. Would removing ineffective alerts work?
      2. Would changing to a different kind of CDS work?
      3. Would changing the visual/interface design of alerts work?

Is there anything else you would like to tell me about alerts or your experience of alert fatigue?
