# Supplementary material for: Experiences of Alert Fatigue and Its Contributing Factors in Hospitals: Qualitative Study
Source: J Med Internet Res. 2026 Feb 19;28:e78676. doi: 10.2196/78676 (PMC12919987; doi:10.2196/78676)
Supplement: Multimedia Appendix 2 [file jmir-v28-e78676-s002.docx]

Multimedia Appendix 2. Proportion of coded references related to alert fatigue mapped to each SEIPS work system domain
