# Supplementary material for: Experiences of Alert Fatigue and Its Contributing Factors in Hospitals: Qualitative Study
Source: J Med Internet Res. 2026 Feb 19;28:e78676. doi: 10.2196/78676 (PMC12919987; doi:10.2196/78676)
Supplement: Multimedia Appendix 3 [file jmir-v28-e78676-s003.docx]

## Strategies discussed by study participants for reducing alert fatigue in practice

| **Technology system** |
| --- |
| Allowing personalisation and customisation of alerts by individuals |
| Automatically populate relevant data in alerts |
| Improve fit of CDS with clinical need (carefully consider whether CDS and which type of CDS is needed to address the issue at hand) |
| Convert interruptive to passive alerts where appropriate |
| Add search functionality within the EHR |
| Improve fit of alerts with workflows, decision-making processes, roles and responsibilities (e.g. tailoring alerts to specific roles) |
| Improve interface design |
| Clear messaging and display (e.g. clear headings, minimal words, use of bolding/colours) |
| Differentiate alerts |
| Require override reasons for highly important alerts |
| Tiering/grading system (and use of colour/other design features to distinguish between different tiers) |
| Increase clinical relevance (including removing non-clinically relevant/redundant alerts) |
| Smarter and less repetitive alerts |
| **Organisational strategies** |
| Monitoring and evaluation |
| Establish committees and working groups to improve alerts |
| Evaluate impact of alerts |
| Seek clinician feedback (particularly JMOs as frequent users of EHR systems) |
| Organisational change |
| Attach a consequence to alerts (e.g. disciplinary action) |
| Change culture and culture of practice (e.g. implement low paging hours, improve general perceptions of alerts within departments) |
| Streamline EHR alerts with other alerts and tasks (i.e. to reduce number of locations doctors need to check for tasks) |
| Organisational support |
| Establish communication channels for questions about alerts (e.g. in EHR itself, WhatsApp channels) |
| Training and education on alert fatigue (to communicate what it is and potential consequences for both patients and clinicians) |
| **Personal strategies** |
| Improve overall health and wellbeing |
| Mental reminders and self-reflection |
| Workarounds (e.g. developing own practices/processes for addressing tasks where alerts are triggered at the wrong point in workflows, setting personal alarms for tasks where important alerts do not exist in the system) |
